# Association of high normal HbA1c and TSH levels with the risk of CHD: a 10-year cohort study and SVM analysis

**DOI:** 10.1038/srep45406

**Published:** 2017-03-27

**Authors:** Hui Li, Ying Cui, Yanan Zhu, Haiying Yan, Wenge Xu

**Affiliations:** 1 Department of Health Administration, Affiliated Central Hospital of Xinxiang Medical University, Xinxiang Central Hospital, Xinxiang, Henan, China

## Abstract

This study aimed to determine the association between the clinical reference range of serum glycated hemoglobin A1c (HbA1c) and thyrotropin (TSH) and the risk of coronary heart disease (CHD) in non-diabetic and euthyroid patients. We examined baseline HbA1c and TSH in 538 healthy participants, and then analyzed the associations and potential value of these indicators for predicting CHD using Cox proportional hazard and support vector machine analyses. During the median follow-up of 120 months, 39 participants later developed CHD. The baseline HbA1c and TSH within the reference range were positively associated with CHD risk. No correlation and interaction were found between the baseline HbA1c and TSH for the development of CHD. Disease event-free survival varied among participants with different baseline HbA1c quintiles, whereas disease event-free survival was similar for different TSH tertiles. The combination of these baselines showed sensitivity of 87.2%, specificity of 92.7%, and accuracy of 92.3% for identifying the participants who will later develop CHD. Relatively high but clinically normal HbA1c and TSH levels may increase the risk of CHD. Therefore, the combination of these indicators can serve as a biomarker for identifying healthy individuals from those who would later develop CHD.

Coronary heart disease (CHD) has one of the highest mortality rates among adults worldwide, especially in developed areas, such as Europe, North America, and China^1^,^2^,^3^. Early prediction combined with subsequent prevention or medical intervention for CHD is the best approach to reduce mortality. Age, gender, family history, blood pressure, cholesterol, and cigarette smoking are the independent risk factors for CHD^3^,^4^. Previous studies successfully used these risk factors to predict the occurrence and development of CHD^5^,^6^,^7^. However, the clinical value of these parameters is often hindered by a number of other interfering factors. Moreover, the prevalence of CHD is also affected by endocrine systems, including glucose homeostasis and thyroid hormone^8^,^9^.

Glycated hemoglobin A1c (HbA1c), a form of hemoglobin, is formed via a non-enzymatic glycation pathway through the exposure of hemoglobin to plasma glucose^10^. Normal levels of glucose produce normal amount of HbA1c^11^. However, the fraction of HbA1c increases in a predictable way when the average amount of plasma glucose increases. This phenomenon serves as a biomarker for the average blood glucose level at 2~3 months before the measurement^12^. In diabetes mellitus, higher amounts of HbA1c, which indicate poor control of blood glucose levels, are firmly associated with the long-term risk of microvascular complications and a series of physical illness, such as cardiovascular disease, nephropathy, neuropathy, and retinopathy^13^,^14^,^15^. Given these favorable performance characteristics, the role of HbA1c as an index of cumulative glycemic exposure in diabetes and cardiovascular risk assessment among non-diabetic individuals has been given increasing attention^16^,^17^. A previous study reported HbA1c as an independent risk factor for the development of atherosclerosis and cardiovascular events, without consideration of diabetes status^18^. Several studies evaluated the ability of baseline HbA1c level in healthy subjects to detect their risk for developing type 2 diabetes mellitus and cardiovascular events^12^,^19^. However, HbA1c measurement is rarely used for predicting CHD in a prospective Chinese cohort with low to average risk.

Thyroid hormones are important regulators of glucose homeostasis and have a definite association with diabetes and CHD. Thyrotropin (TSH), a hormone secreted by the pituitary gland, acts on and stimulates the thyroid gland to produce thyroid hormones. TSH significantly and precisely responds to minor changes in the concentrations of circulating thyroid hormones. However, increasing evidence suggests that TSH exerts extra-thyroidal effects^20^. TSH *in vitro* is capable of stimulating glucose oxidation^21^. Reference range of TSH is linearly and positively associated with body mass index^22^, systolic and diastolic blood pressures^23^, and serum lipid profile levels^24^ with adverse effects on cardiovascular health^25^. Studies involving atherosclerosis patients with normal thyroid function suggest that relatively low TSH levels are associated with severe coronary or carotid atherosclerosis^26^,^27^. An increased risk of cardiovascular disease is present in people with evidence of hypothyroidism^28^ or hyperthyroidism^29^; for TSH in individuals with clinically normal thyroid function, a potential association exists between normal TSH level and risk of CHD.

In summary, HbA1c and TSH play important roles in glucose metabolism and development of CHD. These two parameters are considered biomarkers for this disorder. Hence, we speculated that there may have a link between HbA1c and TSH for developing CHD. However, this link and the ideal value of relatively high baseline HbA1c and TSH in reference range for discriminating individuals who will later develop CHD are poorly understood. In the present study, we aim to detect the association of baseline HbA1c and TSH with the risk of CHD through a 10-year follow-up study involving healthy individuals in a Chinese cohort. In addition, the relationship between baseline HbA1c and TSH was also investigated. Support vector machine (SVM) analysis was conducted to determine whether the combination of baseline HbA1c and TSH in healthy individuals can serve as a predictor for CHD with ideal accuracy and stability.

## Results

### Correlation between baseline HbA1c and TSH and clinical characteristics

**Figure 1:**
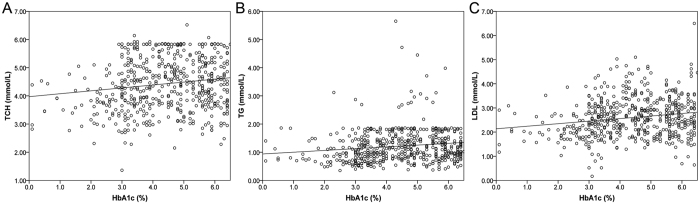
Positive correlations between baseline HbA1c and TCH (**A**), TG (**B**), and LDL (**C**).

The correlations between baseline HbA1c and TSH and serum lipid profiles in type 2 diabetic patients are revealed by previous studies. However, in the healthy examination cohort, we found that HbA1c but not TSH was positively correlated with age (*r* = 0.103, *p* = 0.017), TCH (*r* = 0.150, *p* < 0.001), TG (*r* = 0.145, *p* = 0.001), and LDL (*r* = 0.176, *p* < 0.001) in the baselines. However, the correlation between baseline HbA1c and age of participants was no longer significant after the Bonferroni corrections (*p* < 0.05/8 = 0.00625). The details are shown in Fig. 1. No correlation and interaction were found between baseline HbA1c and TSH (*r* = −0.009, *p* = 0.847; *F* = 0.940, *p* = 0.593).

### Relative risks of CHD by using HbA1c quintiles and TSH tertiles

During the median follow-up of 120 months, 33 participants were withdrawn due to the changes of home address or telephone or other indefinite reasons, 43 participants later developed to diabetes, 9 participants later developed thyroid dysfunction, and 39 participants (12 and 2 also developed diabetes and thyroid dysfunction, respectively) later developed CHD. Twenty-six of the CHD cases were acute coronary syndrome and the remaining cases were chronic coronary artery disease. Baseline HbA1c and TSH were 5.40% ± 1.19% and 2.64 ± 1.00 mIU/L for participants who developed CHD during the follow-up. These values were significantly higher than the 4.32% ± 1.32% and 2.26 ± 1.08 mIU/L for the participants who did not develop CHD (*t* = 4.922, *p* < 0.001; *t* = 2.116, *p* = 0.035).

**Figure 2:**
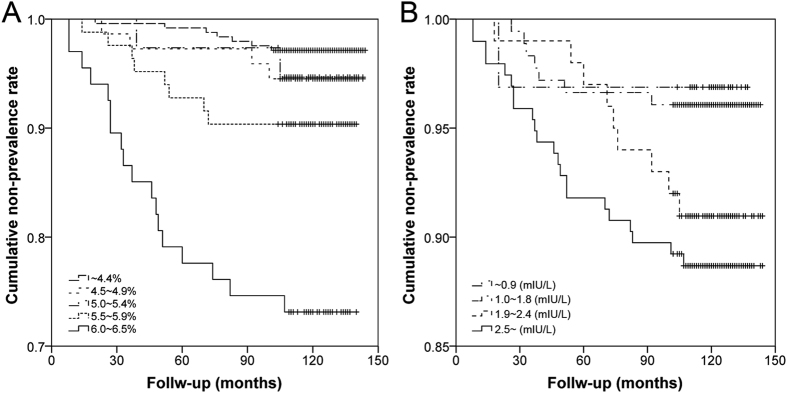
Kaplan-Meier survival curves for 10-year CHD prevalence according to baseline HbA1c (**A**) and TSH (**B**) category.

According to the baseline HbA1c quintiles, a graded risk increase was present in both age-adjusted and multivariable-adjusted models in predicting clinical CHD (*p*’s trend < 0.001, Table 2). The Kaplan–Meier survival curves showed that the CHD event-free survival was significantly different among the participants with different baseline HbA1c quintiles (Log rank *x*^2^ = 49.253, *p* < 0.001, Fig. 2A). Furthermore, according to the baseline TSH tertiles, an increased risk was also presented in both age-adjusted and multivariable-adjusted models in predicting clinical CHD (*p* trend = 0.005 and 0.008, Table 3). However, the Kaplan-Meier survival curves showed that the CHD event-free survival was not significantly different among the participants with different baseline TSH tertiles (Log rank *x*^2^ = 7.200, *p* = 0.062, Fig. 2B). In addition, no obvious association was observed in baseline HbA1c quintiles and TSH tertiles with the type and disease extent of CHD (*p*’s > 0.05, Table 4).

### Discriminating the participants who later developed CHD

**Figure 3:**
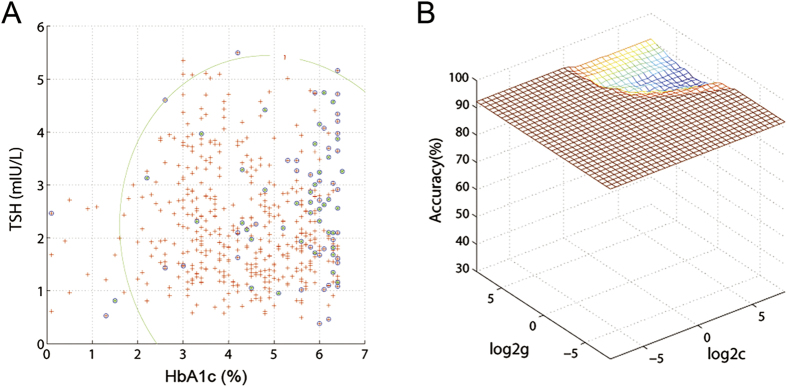
Visualization of classifications in SVM by using the combination of baseline HbA1c and TSH. (**A**) Classified map and (**B**) 3D view of the classified accuracy.

SVM analysis was used to discriminate the participants who later developed CHD. Baseline HbA1c and TSH were used as the training/testing data, whereas the CHD event of the participants was set as the training/testing label (the one who later developed to CHD was set as +1; the one who did not developed CHD was set as −1). Therefore, the combination of baseline HbA1c and TSH had a sensitivity of 87.2% (34/39), a specificity of 92.7% (432/466), and an accuracy of 92.3% (466/505) for discriminating the participants who later developed CHD (the best *C* = 0.0039063, *G* = 0.00396, Fig. 3).

## Discussion

Over the past years, women or elder individuals form a distinct subpopulation within the CHD patients. This observation should be acknowledged in the management and assessment of CHD. In recent clinical analyses, HbA1c and TSH levels have shown greater effect on CHD independent of gender and age. The key finding of the present study is that the baseline HbA1c and TSH within the reference range were positively associated with the increased risk of CHD but were not related to the type or disease extent of this disorder. Individuals with lower baseline HbA1c quintiles present more advantages in the disease event-free survival. Furthermore, to our knowledge, this study is the first to prove that a combination of these baselines holds a sensitivity of 87.2%, a specificity of 92.7%, and an accuracy of 92.3% for discriminating participants who will later develop CHD. In addition, no correlation and interaction were found between baseline HbA1c and TSH for the development of CHD.

Previous results about the role of HbA1c in cardiovascular events are inconsistent. The relationship between the relative high reference range of HbA1c and cardiovascular disease has not been determined^16^. An increase in HbA1c of one percentage point is related to a 20~30% increase in cardiovascular or mortality events^30^; furthermore, the baseline HbA1c ≥6.5% predicts a 10-year risk of this disorder only in healthy women^31^. However, in Caucasian men and especially in women who are 50~75 years old and without diabetes, high HbA1c levels are associated with the increased risk of future non-fatal cardiovascular events, independent of other cardiovascular risk factors^32^. Partly consistent with these conclusions, the present study in healthy examination cohort in Chinese between 31 and 79 years of age revealed that individuals with baseline HbA1c of 6.0~6.5% had 11-fold increase in risk of CHD compared with those with HbA1c below 4.4% when adjusted for age, smoking and drinking history, BMI, blood pressures, history of coronary heart disease in first-degree relatives, and serum lipid profiles. The baseline HbA1c of 6.0~6.5% is a high risk threshold of cardiovascular disorders in multiple populations^33^. In addition, our results also revealed positive correlations between baseline HbA1c and total cholesterol, triglyceride, or low-density lipoprotein cholesterol, which exist in patients with type 2 diabetes^34^. Hence, these results suggest the coordinated roles of HbA1c and lipoprotein in the development of CHD.

A single measurement of low serum TSH in individuals older than 60 years of age is associated with increased mortality from all causes, particularly the mortality due to circulatory and cardiovascular diseases^29^. However, high serum TSH levels are associated with current hypertension and blood pressure^35^. These mechanisms are involved in the effects of thyroid hormones on the cardiovascular system, such as vascular smooth muscle cells, cardiac myocytes, coronary angiogenesis, and renal function^36^,^37^,^38^. Previous studies suggested that differences in thyroid function within the population reference range are differentially associated with CHD risk^39^,^40^. Our current study revealed that the baseline TSH within the reference range is positively associated with the risk of CHD in the healthy examination cohort; individuals with 2.5~5.5 mIU/L of serum TSH level had 4-fold risk of CHD compared with those with 0.3~0.9 mIU/L. However, this serum baseline TSH was not correlated with the lipid profiles, although Xing *et al*.^20^ reported that TSH levels are correlated in a positive linear manner with the total cholesterol and triglyceride levels in Chinese population with newly diagnosed asymptomatic CHD. The difference might be due to the clinical characteristics of the recruited samples, for example, a defined CHD also has abnormal serum total cholesterol and triglyceride levels^41^; however, our samples have relatively normal lipids profiles. Hence, we inferred that the increased risk of CHD within the relative high baseline TSH may result from the intensive effect of thyroid hormones on cardiovascular system.

These evidence suggest a strong association of relatively high normal baseline of HbA1c and TSH with prevalence of CHD that is independent of common coronary risk factors such as age, BMI, blood pressures, and smoking status, which were summarized in previous reviews^3^,^42^. We inferred that several mechanisms independently contribute to this association with the common risk factors. For HbA1c, relatively high HbA1c level reflects the following: 1) continued high blood sugar state^43^, which further leads to dysfunction of vascular endothelial cells; 2) reduced dissociation rate of oxygenation, which increases the oxygen affinity for red blood cells and further leads to tissue hypoxia^44^; and 3) abnormal lipid metabolism and increased oxygen free radicals^45^. These changes are the causes of proliferation of endothelial cells and vascular smooth muscle, clotting of blood vessels, and accelerated development of coronary artery diseases^46^. During the median follow-up of 120 months in 538 healthy individuals, 43 participants later developed diabetes; among which, 12 participants also developed CHD, further suggesting that the mechanism of this association involves abnormal glucose metabolism. For TSH, previous results showed that TSH acts on thyroid gland and targets several other organs and tissues because of the widely expressed receptor^20^,^47^. TSH acts as a physiological regulator in the growth and development of adipocytes by affecting cholesterol homeostasis, including biosynthesis, uptake, and elimination^48^,^49^. Patients with sub-clinical hypothyroidism exhibit increased activity in the plasma platelet-activating factor acetyl hydrolase (PAF-AH) and decreased activity in HDL^50^. Hence, high normal TSH has harmful effects on cardiovascular health through the above lipid metabolic pathways; however, the detailed mechanism on the effects of TSH on CHD remains to be elucidated. In addition, our results revealed that no correlation and interaction exist between HbA1c and TSH. This finding is consistent with the previous report of a seven-year longitudinal study^51^, which suggests that the effects of high normal baseline HbA1c and TSH on the development of CHD may result from different pathological pathways, although the two parameters are related to glucose and lipid metabolisms. Hence, the combination of baseline HbA1c and TSH is valuable for identifying individuals who will later develop CHD.

In general, this study verified the association of baseline HbA1c and TSH with the risk of CHD. This study is the first to combine HbA1c and TSH for predicting CHD in healthy populations. After 505 times of cross-validation, 34 individuals who later developed CHD and 432 who did not develop CHD were correctly classified. Both sensitivity and specificity of this classification were more than 70%, which is an acceptable accuracy for established diagnostic indicators^52^. Baseline HbA1c, TSH, and a combination of these two serum molecules can be applied as potential biomarkers to predict whether healthy individuals will develop CHD in the following years. Furthermore, because the CHD death risk is the second highest specific causes of death^53^ and cardiovascular risk factors are heavily impacting also for all-cause mortality^54^, individuals with relatively high baseline HbA1c or TSH even within reference range need early intervention to prevent disease development. Nevertheless, our sample size was inadequate and could easily lead to false-positive or false-negative results. Targeting this group for preventive interventions is crucial.

## Materials and Methods

### Study design and population

We conducted a prospective study with the individuals who underwent healthy examination in the Xinxiang Central Hospital between June 2003 and October 2005. Individuals who satisfied the following criteria were recruited to the study: (1) men and women aged 18~80 years; (2) without history of and not suffering from diabetes, hyperlipidemia, thyroid dysfunction, and cardiovascular disease; and (3) with available and complete medical reports of healthy examination and contact information. However, individuals were excluded if they have: (1) baseline HbA1c ≥6.5% or significant abnormal baseline TSH; (2) medications that might affect HbA1c and TSH levels and serum lipid profiles; and (3) serious neurologic diseases, psychiatry, renal disorders, or hepatic disorders. A total of 974 individual data during this period were screened from the medical records. Finally, 538 individuals who satisfied the inclusion and exclusion criteria were included in the study. The study was conducted in compliance with the Declaration of Helsinki. All participants wrote and signed their informed consents. The study was approved by the ethics committee of the Xinxiang Central Hospital.

### Baseline data collection

The baseline data including HbA1c, TSH, and serum lipid profiles, as well as the clinical characteristics (age, gender, body mass index, blood pressures, and histories of smoking and drinking, as well as family histories of diseases) were obtained from the medical reports of healthy individuals. HbA1c was estimated using the Tina-Quant turbidimetric inhibition immunoassay (Roche Diagnostics, Indianapolis, Indian), TSH was measured using electrochemiluminescence immunoassay (Elecsys 2010, Roche, Basel, Switzerland), and the lipid profiles were measured using an automatic biochemistry analyzer (OLYMPUS AU5400, Olympus, Japan) and commercial kits. Laboratory reference ranges for HbA1c and TSH were <6.5% and 0.3~5.50 mIU/L, respectively. Clinical baselines of these participants are shown in Table 1.

### Follow-up and endpoint

Long-term follow-up was conducted twice per year through an outpatient interview, telephone call, home visit, or self-reporting of participants. Follow-up time was calculated as the time between the date of the baseline examination and the date of the endpoint event or June 30, 2015. The endpoint of this study was the prevalence of CHD. When the endpoint event occurred, the date of incident and the type (acute coronary syndrome or chronic coronary artery disease) and disease extent (single or multi-vessel) of CHD were recorded.

### Grouping of the subjects

Referenced to the study of Pfister *et al*.^55^, we divided the participants into five categories based on their baseline HbA1c: C1 (HbA1c < 4.4%), C2 (HbA1c 4.5~5.0%), C3 (HbA1c 5.0~5.4%), C4 (HbA1c 5.5~5.9%), and C5 (HbA1c 6.0~6.5%). Furthermore, Teng *et al*.^56^ reported that a baseline TSH of 1.0~1.9 mIU/L is an optimal interval to ensure low incidence of clinical thyroid diseases within five years. The lower limit for normal TSH at 2.5 mIU/L is suggested based on epidemiological studies. Thus, we divided the participants into four categories based on their baseline TSH: G1 (TSH 0.3~0.9 mIU/L), G2 (TSH 1.0~1.8 mIU/L), G3 (TSH 1.9~2.4 mIU/L) and G4 (TSH 2.5~5.5 mIU/L).

### Statistical analysis

SPSS 18.0 software was used for statistical analysis. The relationships between baseline HbA1c and TSH or between the two parameters and other clinical variables were performed using Pearson’s correlation analysis. Bonferroni correction was applied to minimize type I error in the multiple tests. The participants were categorized according to baseline HbA1c quintiles or TSH tertiles. Differences between the tertiles or quintiles were tested with Chi-square (*χ*2) test. Relative risk and 95% confidence interval of baseline HbA1c and TSH for incident CHD event were estimated from the Cox proportional hazard analysis. Tests of linear trends were computed using median values within each of the HbA1c quintiles or TSH tertiles. The first models were adjusted only for age. In the subsequent models, we added the possible confounding factors for the adjustment, including age, smoking and drinking history, BMI, blood pressures, history of CHD in first-degree relatives, and serum lipid profiles. Kaplan-Meier survival curves were plotted and the differences in event-free survival were assessed using the log-rank test for multiple group comparisons. Interaction between HbA1c and TSH for developing CHD was detected through covariance analysis. Two-sided *p* values < 0.05 indicate statistical significance.

### Support vector machine analysis

SVM using the LIBSVM software package (http://www.csie.ntu.edu.tw/~cjlin/libsvm/)^57^ in Matlab was used to examine the possible combination of baseline HbA1c and TSH to discriminate the participants who will later developed CHD. The choice of kernel and parameter *C* is the most important step for the classifier analysis in SVM. Grid search method and Gaussian radial basis function kernels were used for parameter optimization. The “leave-one-out” cross-validation approach, which takes one instance from the original sample as the validation data and leaves the remaining observations as the training data^58^, was applied using the LIBSVM software to obtain the highest sensitivity and specificity. This procedure was repeated 505 times to ensure that each instance of the 505 subjects was used only once as the validation data.

## Additional Information

**How to cite this article**: Li, H. *et al*. Association of high normal HbA1c and TSH levels with the risk of CHD: a 10-year cohort study and SVM analysis. *Sci. Rep.* **7**, 45406; doi: 10.1038/srep45406 (2017).

**Publisher's note:** Springer Nature remains neutral with regard to jurisdictional claims in published maps and institutional affiliations.

## Figures and Tables

**Table 1:** Clinical baselines of the participants.

Characteristic	Values
Sample size (n)	538
Age (Mean ± SD, years)	46.07 ± 9.19
Gender (Male, n, %)	327 (60.80%)
Smoking history (n, %)	96 (17.80%)
Drinking history (n, %)	71 (13.2%)
History of diabetes in first-degree relatives (n, %)	104 (19.3%)
History of coronary heart disease in first-degree relatives (n, %)	66 (12.3%)
BMI (Mean ± SD, kg/m^2^)	22.98 ± 4.52
SBP (Mean ± SD, mmHg)	129 ± 28
DBP (Mean ± SD, mmHg)	80 ± 15
TCH (Mean ± SD, mmol/L)	4.43 ± 0.93
TG (Mean ± SD, mmol/L)	1.21 ± 0.59
HDL (Mean ± SD, mmol/L)	1.28 ± 0.33
LDL (Mean ± SD, mmol/L)	2.60 ± 0.81
HbA1c (Mean ± SD, %)	4.39 ± 1.35
TSH (Mean ± SD, mIU/L)	2.30 ± 1.08
Median follow-up (months)	120

BMI, body mass index; SBP, systolic blood pressure; DBP, diastolic blood pressure; TCH, total cholesterol; TG, triglyceride; HDL, high-density lipoprotein cholesterol; LDL, low-density lipoprotein cholesterol; HbA1c, hemoglobin A1c; TSH, thyrotropin.

**Table 2:** Relative risks of coronary heart disease by using baseline HbA1c quintiles.

	HbA1c quintiles (%)					*P* for trend
	<4.4	4.5~4.9	5.0~5.4	5.5~5.9	6.0~6.5	
Sample size (n)	244	73	38	83	67	
Events (n)	7	4	2	8	18	
Events/1000 person-year	2.8	5.4	5.3	10.1	31.6	
Age-adjusted RR (95% CI)	1.0	2.288 (0.638-8.206)	1.634 (0.324-8.251)	3.250 (1.132-9.333)	11.638 (4.592-29.497)	<0.001
Multivariable-adjusted RR (95% CI)^*^	1.0	1.725 (0.465–6.402)	1.106 (0.207–5.917)	2.900 (0.952–8.832)	11.222 (4.173–30.183)	<0.001

HbA1c, hemoglobin A1c; ^*^adjusted for age, smoking and drinking history, BMI, blood pressures, history of coronary heart disease in first-degree relatives, and serum lipids profiles; RR, relative risk; 95% CI, 95% confidence intervals.

**Table 3:** Relative risks of coronary heart disease by using baseline TSH tertiles.

	TSH tertiles (mIU/L)				*P* for trend
	0.3~0.9	1.0~1.8	1.9~2.4	2.5~5.5	
Sample size (n)	32	178	100	195	
Events (n)	1	7	9	22	
Events/1000 person-year	3.1	3.9	9.2	11.8	
Age-adjusted RR (95% CI)	1.0	1.325 (0.153–11.510)	3.296 (0.386–28.118)	4.375 (0.550–34.776)	0.005
Multivariable-adjusted RR (95% CI)^*^	1.0	1.340 (0.159–11.320)	3.212 (0.389–26.503)	4.006 (0.519–30.925)	0.008

TSH, thyrotropin; ^*^adjusted for age, smoking and drinking history, BMI, blood pressures, history of coronary heart disease in first-degree relatives, and serum lipids profiles; RR, relative risk; 95% CI, 95% confidence intervals.

**Table 4:** Association of the baseline HbA1c and TSH with the type and disease extent of coronary heart disease.

Indicators	Type of CHD (n)			Disease extent of CHD (n)		
	ACS	CCAD	*P* value	Single	Multi-vessel	*P* value
HbA1c (%)						
<4.4	6	1	0.255	1	6	0.162
4.5~4.9	3	1		1	3	
5.0~5.4	2	0		1	1	
5.5~5.9	3	5		4	4	
6.0~6.5	12	6		12	6	
TSH (mIU/L)						
<0.9	1	0	0.648	1	0	0.512
1.0~1.8	4	3		3	4	
1.9~2.4	5	4		3	6	
2.5~5.5	16	6		12	10	

CHD, coronary heart disease; ACS, acute coronary syndrome; CCAD, chronic coronary artery disease; HbA1c, hemoglobin A1c; TSH, thyrotropin.
